# An American Text-Book of Gynecology

**Published:** 1899-12

**Authors:** 


**An American Text-Book of Gynecology.** Edited by J. M. Baldy, M.D. Second Edition. Pp. xxii., 17–718. London: The Rebman Publishing Co. (Ltd.). Philadelphia: W. B. Saunders. 1898.

Of the manner of preparation and the subject matter of the first edition of this work we have previously spoken in terms of praise, and the appearance of the present edition bears testimony to a general appreciation which is well bestowed.

Although the work has been rearranged to some extent, there is no need to criticise it in detail. The main alterations consist in fuller treatment of the chapters on the bladder, urethra and ureters, hysterectomy, and plastic operations. The teachings of its pages are thoroughly sound and well abreast of the times, and we can confidently recommend the book as a guide to the routine work of the gynæcologist. The illustrations as a whole are good; but there are still some, such as Figs. 26 and 27, which might without loss be omitted in a future issue.

